# *ROS1* rearrangements in lung adenocarcinoma: prognostic impact, therapeutic options and genetic variability

**DOI:** 10.18632/oncotarget.3387

**Published:** 2015-03-25

**Authors:** Matthias Scheffler, Anne Schultheis, Cristina Teixido, Sebastian Michels, Daniela Morales-Espinosa, Santiago Viteri, Wolfgang Hartmann, Sabine Merkelbach-Bruse, Rieke Fischer, Hans-Ulrich Schildhaus, Jana Fassunke, Martin Sebastian, Monika Serke, Britta Kaminsky, Winfried Randerath, Ulrich Gerigk, Yon-Dschun Ko, Stefan Krüger, Roland Schnell, Achim Rothe, Cornelia Kropf-Sanchen, Lukas Heukamp, Rafael Rosell, Reinhard Büttner, Jürgen Wolf

**Affiliations:** ^1^ Center for Integrated Oncology Köln Bonn, Cologne, Germany; ^2^ Lung Cancer Group Cologne, Department I for Internal Medicine, University Hospital of Cologne, Cologne, Germany; ^3^ Institute of Pathology, University Hospital of Cologne, Cologne, Germany; ^4^ Pangaea Biotech, Quirón Dexeus University Hospital, Barcelona, Spain; ^5^ Institut d'Investigació en Ciències de la Salut, Germans Trias i Pujol, Badalona, Spain; ^6^ Instituto Oncológico Dr Rosell, Quirón Dexeus University Hospital, Barcelona, Spain; ^7^ Gerhard-Domagk-Institute of Pathology, University Hospital of Münster, Münster, Germany; ^8^ Institute of Pathology, University Hospital of Göttingen, Göttingen, Germany; ^9^ Department of Hematology/Oncology, University Hospital of Frankfurt, Frankfurt, Germany; ^10^ Department for Pulmonology and Thoracic Oncology, Lung Clinic Hemer, Hemer, Germany; ^11^ Clinic for Pneumology and Allergology Center for Sleep Medicine and Respiratory Care, Bethanien Hospital, Solingen, Germany; ^12^ Thoracic Centre, Malteser Hospital Bonn/Rhein-Sieg, Bonn, Germany; ^13^ Johanniter Hospital, Evangelical Clinics of Bonn, Bonn, Germany; ^14^ Clinic for Pneumology/Allergology/Sleep Medicine and Respiratory Care, Florence-Nightingale-Hospital, Düsseldorf, Germany; ^15^ Practice for Internistic Oncology and Hematology, Frechen, Germany; ^16^ Practice for Hematology and Oncology Mainka/Dietze/Rothe, Cologne, Germany; ^17^ Department II for Internal Medicine, University Hospital of Ulm, Ulm, Germany; ^18^ Cancer Biology and Precision Medicine Program, Catalan Institute of Oncology, Hospital Germans Trias i Pujol, Badalona, Spain; ^19^ Molecular Oncology Research (MORe) Foundation, Barcelona, Spain

**Keywords:** non-small cell lung cancer, ROS1, prognosis, chemotherapy, lung cancer

## Abstract

**Background:**

While recent data show that crizotinib is highly effective in patients with *ROS1* rearrangement, few data is available about the prognostic impact, the predictive value for different treatments, and the genetic heterogeneity of *ROS1*-positive patients.

**Patients and Methods:**

1137 patients with adenocarcinoma of the lung were analyzed regarding their *ROS1* status. In positive cases, next-generation sequencing (NGS) was performed. Clinical characteristics, treatments and outcome of these patients were assessed. Overall survival (OS) was compared with genetically defined subgroups of *ROS1-*negative patients.

**Results:**

19 patients of 1035 evaluable (1.8%) had *ROS1-*rearrangement. The median OS has not been reached. Stage IV patients with *ROS1-*rearrangement had the best OS of all subgroups (36.7 months, *p* < 0.001). 9 of 14 (64.2%) patients had at least one response to chemotherapy. Estimated mean OS for patients receiving chemotherapy and crizotinib was 5.3 years. Ten patients with *ROS1*-rearrangement (52.6%) harbored additional aberrations.

**Conclusion:**

*ROS1-*rearangement is not only a predictive marker for response to crizotinib, but also seems to be the one of the best prognostic molecular markers in NSCLC reported so far. In stage IV patients, response to chemotherapy was remarkable high and overall survival was significantly better compared to other subgroups including *EGFR*-mutated and *ALK-*fusion-positive NSCLC.

## INTRODUCTION

Non-small cell lung cancer (NSCLC) is still the leading cause of cancer-related death in the western world [1]. Nevertheless, the identification of therapeutically targetable oncogenic driver aberrations has led to an improvement on the clinical outcome of genetically defined subgroups of patients, like those harboring a sensitizing mutation within the epidermal growth factor receptor gene (*EGFR*) treated with EGFR-directed tyrosine kinase inhibitors (TKIs) or with rearrangements of the *ALK* oncogene treated with specific TKIs [2–6]. Additionally, improved molecular diagnostics led to a genomics-based classifications of NSCLC [7].

Chromosomal rearrangements involving the *ROS1* gene (*c-ros oncogene 1*) have recently been identified and described in 1–2% of patients with lung cancer [8, 9]. *ROS1* (chromosome 6q22) encodes a receptor tyrosine kinase which belongs to the insulin receptor family, with downstream signaling via the MAPK pathway through phosphorylation of RAS [10]. In lung cancer, *ROS1* fusion partners include *FIG*, *CD74*, *SLC34A2* and *SDC4*, which lead to oncogenic transformation and constitutive kinase activity in cell culture and/or *in vivo* [8, 11, 12]. So far, no ligand for the ROS1 tyrosine kinase has been identified.

Preclinical data suggest that ROS1 can be targeted by ALK inhibitors due to highly similar tyrosine kinase domains [13]. These findings together with the clinical notion that the cohort of *ROS1*-rearranged patients share features with *ALK*-rearranged patients led to the discovery that crizotinib is an effective treatment option with high response rates [9]. Nevertheless, few is known about the prognostic value, the clinical presentation, the predictive value for different therapy regimens, and the genetic heterogeneity in terms of multiplex-sequencing of patients harboring *ROS1* rearrangement.

We set out this study in order to genetically and phenotypically identify patients with *ROS1*-rearranegments as part of an international, oligocentric prospective phase II trial to assess the response rates of patients with *ROS1*-rearrangement treated with crizotinib (clinicaltrials.gov, NCT02183870), within a molecular screening network. The aim of this study was to detect the prevalence and incidence of *ROS1* rearrangement in these patients, to analyze specific features of *ROS1* rearrangement detection by fluorescence in situ hybridization (FISH), to describe their clinical and pathological characteristics, to assess co-occurring mutations measured by high-standard techniques (next-generation sequencing [NGS]), and to compare stage IV *ROS1*-positive patients with stage IV patients with other defined genetic aberrations regarding survival (i.e. *EGFR, EML4-ALK, FGFR1, KRAS*).

## RESULTS

### ROS1-rearrangement patterns

The majority of the samples were biopsy specimens (i.e. core needle biopsies, ultrasound-guided transbronchial biopsies and cytology specimens). Of all *ROS1*-rearranged cases, only 1 sample was material from total tumor resection lobectomy. None of the *ROS1* rearranged cases was a cytology specimen (i.e. blocked material from fine needle aspiration).

**Figure 1:**
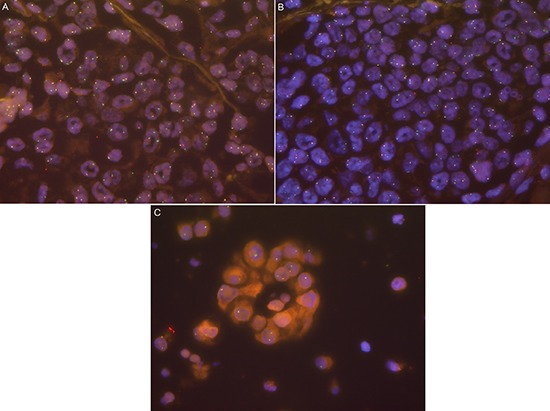
**(A)** *ROS1-*rearranged case with clear split signals and additional green signals**(B)** *ROS1-*rearranged case with primarily additional green signals and few clear split signals. **(C)** *ROS1*-rearranged case with only additional green signals indicating an unbalanced translocation.

*ROS1* status was evaluable in 1035 out of 1137 (91.0%) patients, whereof 19 patients (1.8%) had a *ROS1* rearrangement. *ROS1* signals were homogeneously distributed in all analyzed tumors. The amount of cells showing aberrant signals ranged between 23% and 100% (mean 66%, median 67%). In all rearranged cases we observed an even signal distribution over the entire tumor with no “hot spot” areas. However, among different rearranged tumors, we observed a certain variation in the signal patterns. Some tumors showed only additional 3′ signals with no or few split signals, indicating an unbalanced translocation. In contrast, other tumors showed a homogenous split signal pattern in all tumor cells (see Figure 1).

### Clinical presentation

10 patients were male and 9 patients female. A summary of the clinical characterization is given in Table 1. The median age at diagnosis was 60 years (range, 26–87). In one patient, there were partially neuroendocrine patterns in the tumor. The majority of patients presented with stage IV disease at diagnosis (*n* = 14). 13 patients (68.4%) were never-smokers, whereas 3 were active smokers and 3 patients had a smoking history. Patients with smoking history had a median of 40 pack-years (range, 30–45). The clinical and pathological presentation of each patient is listed in Table 2.

**Table 1:** Clinical characteristics of patients with ROS1-rearrangement (*n* = 19)

Characteristics	Number of patients	Years	%
**Age at diagnosis** *Mean* *Standard deviation* *Median* *Range*	19	58.6 14.8 60 26–82	100
**Gender** *Women* Men	9 10		47.4 52.6
**Smoking** *Never* *Former* *Current*	13 3 3		68.4 15.8 15.8
**UICC tumor stage** *I* *II* *III* *IV*	2 0 3 14		10.5 0 15.8 73.7

**Table 2:** Line listing of the patients

Pat ID	Gender	Age (years)	Initial stage	ROS1 transl. (%)	Additional genetic aberration	Further analyzed	Pack-years	Systemic therapy lines – CTX	BR CTX	Best response CTX under	BR crizotinib	Overall survival (months)
**02**	m	69	IV	100	-	ALK FISH	45	2	n/a	n/a	n/a	36.72
**03**	m	55	IV	100	*TP53* P72R	NGS	0	8	PR	CARBO/GEM/BEV (3x)	PR	63.64*
**04**	m	62	IV	23	-	NGS	30	1	SD	CARBO/PEM	n/a	6.79*
**05**	m	50	IV	25	*EGFR* P848L, *TP53* P72R	NGS	0	1	n/a	n/a	n/a	1.41*
**06**	m	56	IV	25	-	NGS, HER2 FISH	0	1	PR	CIS/PEM	n/a	12.20*
**07**	m	78	IIIB	50	*TP53* E204*, *TP53* P72R	NGS	0	1	PD	n/a	n/a	4.13
**08**	f	26	IV	62	*TP53* K305*	NGS, HER2 FISH	0	2	PD	n/a	n/a	2.95
**09**	m	52	IA	76	-	NGS	30	n/a	n/a	n/a	n/a	16.59*
**10**	f	61	IIIB	83	*TP53* P72R	NGS	0	1	PR	(RCTX)	n/a	12.75*
**11**	m	31	IV	100	-	NGS	0	1	PR	CIS/PEM/BEV	n/a	21.25*
**12**	f	87	IV	55	*MET* R988C	NGS, HER2 FISH	0	1	PR	GEM	n/a	3.77*
**13**	f	62	IA	65	*BRAF* G469S	NGS	40	n/a	n/a	n/a	n/a	41.64*
**14**	f	69	IV	67	*TP53* P72R	NGS	0	1	PR	CARBO/PACLI/BEV	PR	13.34*
**15**	f	54	IIIA	95	-	ALK, RET FISH	0	n/a	n/a	n/a	n/a	3.08
**16**	f	50	IV	96	-	NGS	0	5	PR	GEM/CET, DOCE/CET	PR	78.59*
**17**	m	78	IV	74	*BRAF* c.1742–1G>T	NGS	40	1	SD	PEM	n/a	3.02*
**18**	m	62	IV	26	*MAP2K1* K57N, *TP53* R248L	NGS	40	1	SD	PEM	n/a	5.11*
**19**	f	51	IV	96	-	EGFR, KRAS, BRAF, ALK	0	5	PR	DOCE	PR	33.25*
**20**	f	60	IV	70	-	EGFR, ALK, KRAS, BRAF, RET	0	3	PR	CIS/PEM	PR	27.67

Abbreviations: m = male, f = female; NGS = next-generation sequencing, FISH = flourescence in situ hybridization; CTX = chemotherapy, BR = best response, PR = partial response, SD = stable disease, PD = progressive disease; CARBO = carboplatin, GEM = gemcitabine, BEV = bevacitzumab, PEM = pemetrexed, RCTX = combined radio-chemotherapy, PACLI = paclitaxel, CET = cetuximab, DOCE = docetaxel.

* for OS: ongoing. NGS: panel with 102 amplicons and 14 genes.

### Co-occurring mutations detected by NGS

15 of the 19 (78.9%) detected patients could further be analyzed by NGS. In 7 (46.7%), aberrations within the *TP53* gene were detected: 5 patients (33.3%) had the P72R-polymorphism, whereof one patient had also a E204* mutation, and one patient had a K305* as the only additional aberration. One patient had a R248L mutation co-occurring with a *MAP2K1* missense mutation (K57N). Of the P72R patients, one had an *EGFR* mutation in exon 21 (P848L). All *TP53* alterations were either known polymorphisms or inactivating and truncating mutations.

2 out of 17 patients analyzed with NGS or by Sanger sequencing (11.8%) had *BRAF* mutations (G469S and an intron mutation c.1742–1 G > T, p.? which has not been described yet). One patient (6.7%) showed a *MET* mutation (R988C), located within the juxtamembrane region. Taken together, in 10 of 15 (66.7%) patients analyzed by NGS, further genetic aberrations were detected (see Table 2). Both *BRAF* mutations occurred in patients with a smoking history.

In all four patients without NGS analysis the *ALK* status analysis was performed using FISH. None of them harbored a translocation. *RET* status was negative in the two patients analyzed. Sanger sequencing did not reveal any other genetic aberrations.

### Survival analysis

**Figure 2:**
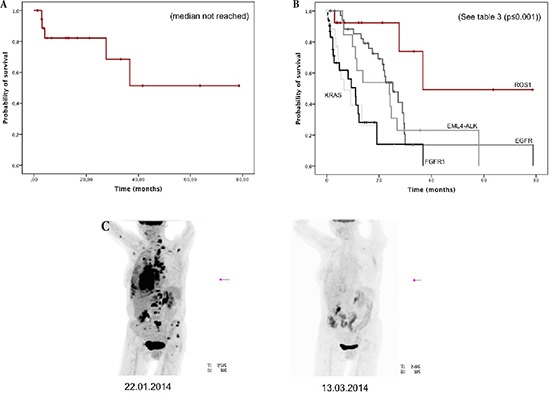
**(A)** Overall survival of all patients with ROS1-rearrangement (*n* = 19)**(B)** Overall survival of stage IV with ROS1-rearrangement (*n* = 14) and comparison with other genetically defined stage IV subgroups (*n* = 115). **(C)** Example of impressive metabolic response in a patient with ROS1-rearrangement treated with crizotinib at baseline and after two months of therapy.

Median follow-up time (see supplementary data) was 16.6 months (95% CI, 9.3–23.9 months). For patients with ROS1-rearrangement, the median OS was not reached (see Figure 2A). The estimated mean survival time was 51.1 months (95% CI, 32.1–70.0 months).

Patients with stage IV (*n* = 14) were further compared with 115 stage IV patients comprising 38 patients with *EGFR* mutation treated with erlotinib and/or gefitinib and/or afatinib (+/− cetuximab), 13 patients with *ALK* rearrangement treated with crizotinib and/or ceritinib, 32 patients with *KRAS* mutations and 32 patients with squamous-cell carcinoma and *FGFR1* amplification (see Supplementary Figure 1). Survival for the *ROS1*-positive patients was significantly better than in the comparison group (36.7 months vs 17.5 months, *p* = 0.005). Median overall survival for the *ROS1*-patients who did not receive crizotinib treatment (*n* = 9) was 36.7 months, whereas the median for the five patients receiving crizotinib has not been reached (estimated mean OS, 65.9 months [95% CI, 44.3 – 87.5 months], see Supplementary Figure 2). Due to the small sample-size and the low prevalence of events, no statistical significance between *ROS1* stage IV patients with or without crizotinib treatment (*p* = 0.279, see supplement) could be found.

Compared with genetically predefined subgroups, patients with *ROS1*-rearrangement had the best OS (*p* ≤ 0.001, see Figure 2B and Table 3). OS was significantly prolonged compared to *EGFR*-mutated patients (36.7 vs. 25.3 months, *p* = 0.047) and to *ALK*-rearranged patients (36.7 vs. 23.9 months, *p* = 0.026), both treated with target-specific therapy. Taken both, *EGFR*-positive and *ALK*-positive patients together, their OS was 24.2 months (95% CI, 20.9–27.5 months) and remained significantly shorter than for *ROS1*-rearranged patients (*p* = 0.033).

**Table 3:** Summary of the subgroups used for comparison of OS

	ROS1	EGFR	EML4-ALK	KRAS	FGFR1
Patients (n)	14	38	13	32	32
Median age (range)	58 (25–87)	70 (30–86)	42 (28–70)	64 (35–83)	68 (47–80)
Median OS months (95% CI)	36.7 (n. a.)	25.3 (19.3–31.3)	23.9 (8.9–38.9)	6.6 (1.2–12.0)	11.0 (5.6–16.4)

### Treatment outcomes

Of the 14 stage IV patients, 12 were evaluable for outcome analysis (one had an individual treatment approach with combining systemic therapy with local procedures, one was lost to follow-up after irradiation of cerebral metastasis). Two stage IIIB patients were taken into analysis. The 14 patients received between one and 8 chemotherapy lines respectively (median = 1, see Table 2). Of them, 9 patients (64.3%) had at least one radiological response to chemotherapy, 3 (21.4%) had stable disease as best outcome and 2 (14.3%) did not respond to chemotherapy (see Supplementary Figure 3A). Table 2 shows the different outcomes of the patients. Both patients not responding to treatment had additional truncating *TP53* mutations, possibly indicating that p53 inactivation might be a surrogate marker for chemoresistance of *ROS1*-positive tumors. Due to the small number of patients without benefit from chemotherapy, we did not check for a correlation of the percentage of translocated tumor cells and clinical outcome.

Supplementary Table 1 lists the used regimen and their outcomes. Noteworthy, while platinum/pemetrexed combinations showed high response rates with four out of five times used, the commonly used first-line therapy of platinum/paclitaxel only led to one response in six applications (see Supplementary Figure 3B and Supplementary Table 1). In all cases, treatment with erlotinib led to primary progression (*n* = 3). In three cases, progression developed under bevacizumab maintenance therapy after initial response (data not shown).

Five patients received crizotinib treatment; all of them responded impressively to monotherapy (see Figure 2C). So far only one patient died under therapy, whereas the remaining four are still ongoing under therapy.

## DISCUSSION

To our knowledge, this study is the first analysis of Caucasian *ROS1*-rearranged patients demonstrating an OS advantage compared to other patients with NSCLC, even when compared to targeted-treated *EGFR*-mutated and/or *ALK*-rearranged patients. Further, this analysis suggests a potential benefit of chemotherapy for this subgroup of NSCLC patients regarding OS and response and stays in line with recent reports of the high efficacy of crizotinib treatment for these patients [9, 14–17]. Taken together, due to high response rates under both “classic” cytotoxic therapy and targeted therapy with crizotinib for stage IV patients *ROS1*-rearrangement seems to repesent one of the best prognostic factors in NSCLC reported so far. Estimated mean survival times for all patients are in the range of years, and until today, no median OS has been reached, neither for the entire population nor for the stage IV patients receiving crizotinib.

Due to its exploratory character, this analysis suffers some limitations: The number of patients described is small, and no power calculation has been performed a priori. By nature, all the analyses presented here were retrospective, and pooling the treatment regimens and the resulting response rates is not comparable to outcomes in prospective clinical trials. Nevertheless, given the low frequency of 1.8% in 1035 screened patients and the very recent discovery of this aberration, such retrospective analyses are needed to gain more insights in the clinical course of these patients. Clinical trials to evaluate the efficacy of crizotinb treatment prospectivly for patients with *ROS1*-rearrangement are ongoing, and these data will be available in the near future.

Importantly and in some contrast to published literature [9], our data show that *ROS1*-rearrangements are not mutually exclusive with other transformation-associated genetic aberrations, as the majority of the patients presented with additional mutations. Beside mutations in *BRAF*, *MET*, and *MAP2K1*, we found a large variety of mutations affecting *TP53*. In two cases, truncating mutations of *TP53* co-occurred with progressive disease under chemotherapy, whereof one patient, a 26-year old female patient, also presented with a partial neuroendocrine histology. Analysis of larger series of *ROS1* positive patients in the future will elucidate whether these additional aberrations might play a role in the development of resistance.

In *ALK*-rearaanged patients, pemetrexed is an efficient treatment choice [18]. While the efficacy of crizotinib in *ROS1*-rearranged patients has been recently shown [16], our dataset also suggests a benefit for pemetrexed-containing regimens in these patients. Therefore, drug development comprises both genetic subgroups [19]. Surprisingly, paclitaxel-containing platinum therapy seems to underperform in this patient group. Although the interpretation of this observation clearly is limited by the low patient number, it will be interesting to see if the choice of the primary platinum-based chemotherapy regimen has an impact on outcome.

Taken together, *ROS1*-rearranged patients represent a unique subgroup of NSCLC patients, with a relatively good prognosis, a remarkable good outcome under different regimens of chemotherapy and dramatic responses under crizotinib. Future analysis will reveal more insights regarding the role of additional genetic aberrations in acquired resistance and differences in the efficacy of distinct chemotherapeutic regimens.

## METHODS

### Patients

This study was performed within a collaborative health care provider network for comprehensive molecular diagnostics of lung cancer in Cologne, Germany (Network Genomic Medicine [NGM], based at the Center for Integrated Oncology Köln-Bonn [CIO], Cologne, Germany), where samples of 1024 patients were analyzed, and in Barcelona, Spain, where 113 samples were screened for *ROS1*-status. The screening process was performed in order to detect patients who might participate in the aforementioned clinical trial. The trial has been approved by local auhorities and the responsible ethics committees. Screening procedures were conducted in concordance with the local ethical guidelines and were reviewed by the institutional ethics committee. All patients consented to be contacted after diagnosis and to provide information about the clinical history and outcome. Insight into their medical records was obtained. The present study covers a timeframe from August 2012 to April 2014. There was no preselection of patients regarding stage or clinical presentation.

Within the same timeframe, we collected data from patients without *ROS1*-rearrangement, who provided written informed consent to have their data analyzed, but with other defined aberrations. As *ROS1*-rearrangement most probably occurs in patients without smoking history [9], and given the fact that NSCLC in never-smokers differs heavily from those of smokers in terms of mutational variability [20], we chose *ALK*-rearranged and *EGFR*-mutated patients with similar smoking habits as the best fitting control group. For both groups, patients were only taken into analysis if they had received treatment with at least one therapy-line with TKIs (i. e., erlotinib, gefitinib, afatinib, AZD9291 for EGFR, crizotinib and/or ceritinib for EML4-ALK), representing the subgroups of NSCLC patients so far with the best overall survival (OS) in stage IV NSCLC [7]. We also analyzed patients with *KRAS* mutations to provide a group without any targeted therapy option, and *FGFR1*-amplified squamous-cell carcinoma patients as a comparator smoking associated lung cancer [21].

### Samples and immunohistochemistry

Tumor tissue was fixed in buffered formalin and embedded in paraffin blocks. All primary diagnoses were reviewed by two experienced pathologists. Morphologic features, e.g. size of nuclei, nucleus/cytoplasm ratio and chromatin structure were evaluated on haematoxylin-eosin slides. To confirm the diagnosis ancillary immunohistochemical stainings were made, e.g. cytokeratins (CK5/6, CK7, p40) as well as TTF1 (thyroid transcription factor 1). Tumor diagnoses were made in accordance to the current WHO classification system [22].

### FISH assay

For FISH, three to four μm tissue sections were mounted on sialinized slides and hybridized overnight with the Zyto*Light©* SPEC *ROS1* Dual Color Break Apart Probe (ZytoVision, Bremerhaven, Germany). The 3` *ROS1* probe was labeled with ZyGreen™ and the 5` *ROS1* probe was labeled with ZyOrange™. An exact protocol about the procedures is given in the supplementary file.

Tumors were defined as *ROS1* rearranged when having ≥ 20% of tumor cells harboring aberrant signals.

### Next-generation sequencing (NGS)

A more detailed protocol of tissue preparation for NGS is added as a supplementary file. Targeted next generation sequencing (NGS) was performed on all FFPE samples. Isolated DNA (< 0.5 – 200 ng/μl) was amplified with an in-house specified, customized Ion AmpliSeq Primer Pool (Lifetechnologies, Carlsbad, USA). The panel comprises 102 amplicons of 14 different genes. PCR products were ligated to adapters and enriched for target regions using the Ion AmpliSeq Panel^TM^ Library kit according to manufacturer's instructions (Lifetechnologies). The generated libraries were pooled equimolarly for amplicon sequencing to a concentration of 3 nM of each sample to counterbalance differences in sample quality. Sequencing was performed on the Illumina MiSeq benchtop sequencer (Illumina, San Diego, USA). Results were visualized in the Integrative Genomics Viewer (IGV) and then manually analyzed. A 5% cutoff for variant calls was used and results were only interpreted if the coverage was > 200.

### Clinical parameters

Age, gender, and tumor stage at diagnosis according to the UICC classification were assessed. Smoking status, medical history regarding past cancer therapies, and outcome to treatments were analyzed. For smoking status, pack-years were annotated. The following qualitative attribution was assessed: Patients with less than 100 cigarettes in their lifetime were considered as never smokers, patients with more than 100 cigarettes, who quit smoking at least one year before first diagnosis of lung cancer were considered former smokers, and patients with a smoking history of more than one pack-year who continued smoking for a period shorter than one year before diagnosis were considered current smokers.

### Statistics

Qualitative variables were summarized by count and percentage, quantitative variables (i.e. age) by mean, standard deviation, median and range. Distribution of time to event was described by the Kaplan-Meier curve and compared between groups by the log-rank test, giving the 95% confidence interval (95% CI). Association of qualitative variables was tested for by chi-square or Fisher's exact test, contingent on distributional assumptions. Overall survival (OS) was defined as the time period from the date of first diagnosis until death. Patients who were still alive at the data cut-off were censored.

## METHODS


