# Supplementary material for: ROS1 rearrangements in lung adenocarcinoma: prognostic impact, therapeutic options and genetic variability
Source: Oncotarget. 2015 Mar 25;6(12):10577–85. doi: 10.18632/oncotarget.3387 (PMC4496376; doi:10.18632/oncotarget.3387)
Supplement: Supplementary file 1 [file oncotarget-06-10577-s001.pdf]

METHODS

FISH procedures

Deparaffinization, protease treatment, and washes were performed on the half-automated VP2000 processor system (Abbott Molecular, Wiesbaden, Germany). After pretreatment, the slides were denatured in the presence of up to 10 µl probe for 5 min at 75°C and hybridized at 37°C overnight. Post-hybridization SSC washes were performed at 72°C and the slides stained with DAPI before analysis. Normal tissue including vessels, fibroblasts, lymphocytes or non-tumor lung tissue served as internal positive control. Cases were only further evaluated if control tissue nuclei displayed one or two clearly distinct signals of each color. Tumor tissue was scanned for potential tumor heterogeneity by using a 63x objective and appropriate filter sets (DM5500 fluorescent microscope; Leica). If *ROS1* signals showed a homogenous distribution, random areas were used for counting the signals. One hundred non-overlapping contiguous tumor cell nuclei from several different areas, resulting in a total of 100 nuclei, were individually evaluated with the 100x or 63x objectives. An aberrant cell was defined by a cell showing at least one orange/green fusion signal and one or more separate orange and one or more separate green split signals or at least on orange/green fusion signal and one or more isolated 3' (green) signals. *ROS1* rearrangement cut off value was defined on a subset of 100 healthy human tissue samples.

Next generation sequencing (NGS)

All samples were fixed in neutral-buffered formalin prior to paraffin embedding (FFPE-samples). On a haematoxylin-eosin stained slide tumor areas were selected by a pathologist (R.B., A.S.) and DNA was extracted from corresponding unstained 10 µm thick slides by manual micro-dissection. The DNA was isolated by automated extraction using the Maxwell 16 System (Promega, Mannheim, HGER) following the manufacturer's protocols. Quality and quantity of isolated DNA was assessed by agarose gel electrophoresis, by a Nanodrop 2000c spectrophotometer (PeqLab, Erlangen, GER) or in the case of next generation sequencing with the Qubit® Fluorometer (Life Technologies, Carlsbad, USA). Targeted next generation sequencing (NGS) was performed on all FFPE samples. Isolated DNA (<0.5 – 200 ng/µl) was amplified with an in-house specified, customized Ion AmpliSeq Primer Pool (Lifetechnologies). The panel comprises 102 amplicons of 14 different genes (see below). PCR products were barcoded and ligated to adapters and enriched for target regions using the Ion AmpliSeq Panel™ Library kit according to manufacturer's instructions (Lifetechnologies). The generated libraries were equimolarly pooled for amplicon sequencing to a concentration of 3 nM of each sample to counterbalance differences in sample quality. Sequencing was performed on an Illumina MiSeq benchtop sequencer (Illumina, San Diego, USA). Results were visualized in the Integrative Genomics Viewer (IGV) and manually analyzed. A 5% cutoff for variant calls was used and results were only interpreted if the coverage was >200.

The following regions were analyzed:

KRAS Exon 2 and 3
PIK3CA Exon 9 and 20
BRAF Exon 11 and 15
EGFR Exon 18–21
HER2 Exon 19 and 20
NRAS Exon 2 and 3
DDR2
TP53 Exon 4–8
ALK Exon 21–25
CTNNB1 Exon 3
MET Intron 13/14, Exon 14
AKT1 Codon E17 (Exon 3 or 4)
PTEN
MEK1 (MAP2K1) Exon 2

Treatment outcomes (response evaluation)

Outcomes were assessed locally by the investigators using Response Evaluation Criteria in Solid Tumors (RECIST) 1.1 if possible (Eisenhauer et al., Eur J Cancer, 2009). In cases where positron-emission-tomography (PET) data was used to evaluate the response, PERCIST guidelines were used (Wahl et al., J Nucl Med, 2009).

Follow-up time was assessed using "reverse" Kaplan-Meier statistics, i.e. death was considered censored, whereas ongoing patiens were considered an event (Schempers et al., Controlled Clin Trials, 1996).

SUPPLEMENTARY FIGURES AND TABLE

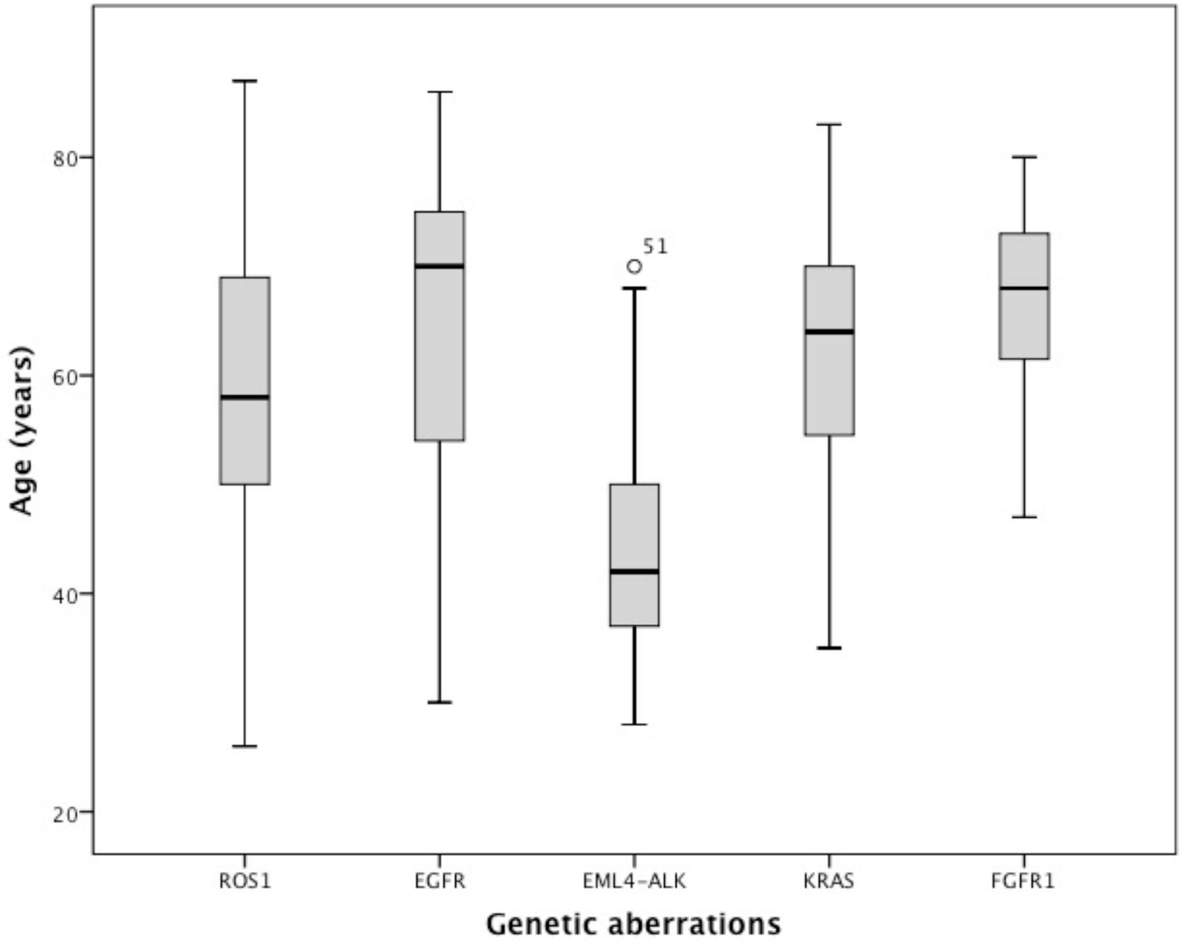

Supplementary Figure 1: Age distribution in the genetically different subgroups.

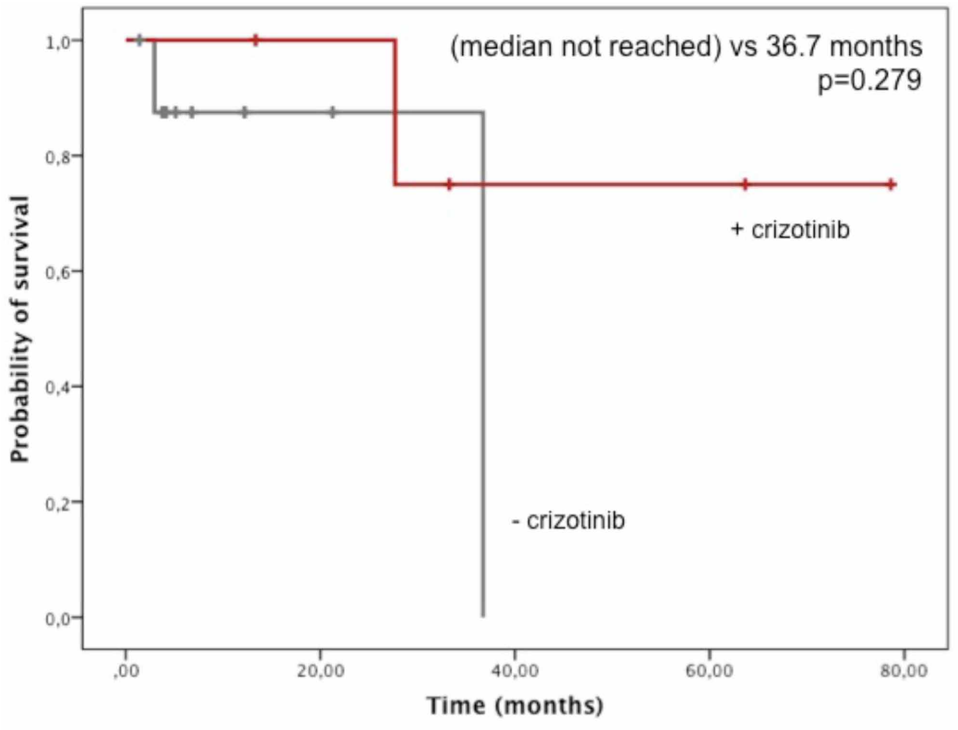

Supplementary Figure 2: Overall survival of stage IV patients with ROS1-rearrangement regarding exposure to crizotinib (n = 5) or crizotinib-naive (n = 9).

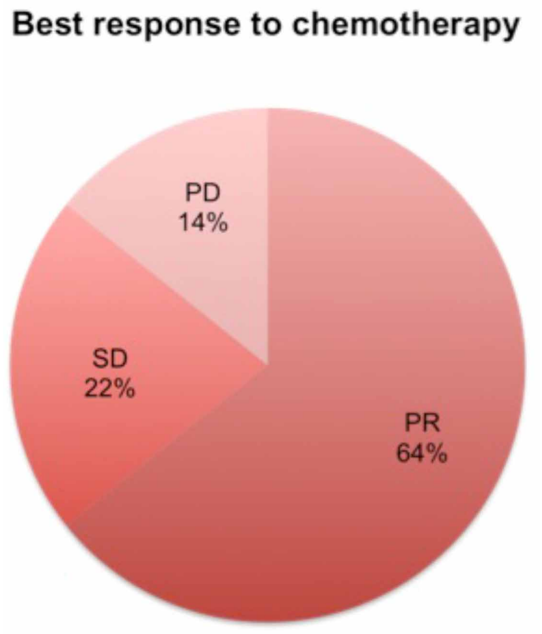

Supplementary Figure 3: (A) Best response for the individual patients of at least one regimen of chemotherapy.

(*Continued*)

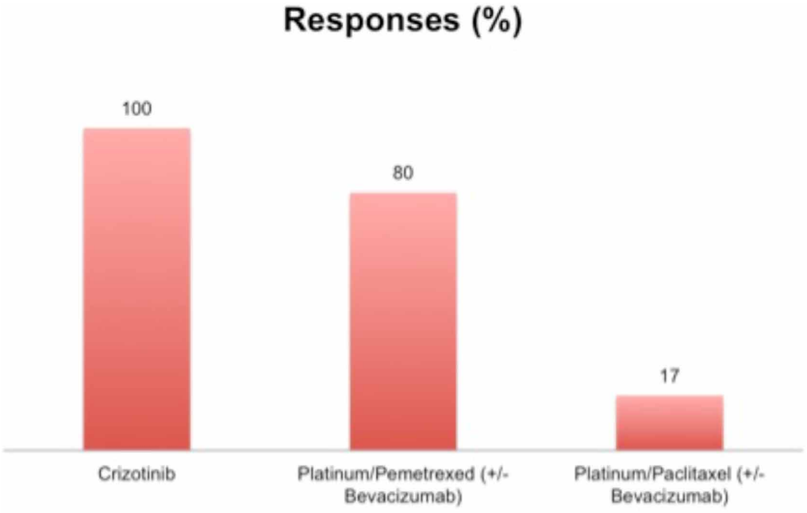

Supplementary Figure 3: (*Continued*) (B) Comparing responses under crizotinib (n = 5), pemetrexed-containing platinum therapy (n = 5) and paclitaxel-containing platinum therapy (n = 6).

Supplementary Table 1: Pooled chemotherapies and their best outcomes

Therapy	PR	SD	PD
Platinum/Pemetrexed (+/− Bevacizumab)	4	0	1
Pemetrexed mono	0	3	1
Platinum/Gemcitabine (+/− Bevacizumab)	1 (3x in one patient)	1	0
Gemcitabine mono	2**	1*	0
Platinum/Paclitaxel (+/− Bevacizumab)	1	0	5
Docetaxel	2**	0	0
Platinum/Vinorelbine	0	0	1
Vinorelbine mono	0	1*	2
Erlotinib	0	0	3
Sunitinib	0	0	1
Cisplatin/Etoposide	0	0	1
Crizotinib	5	0	0

*Gemcitabine + Vinorelbine
**+ Cetuximab in one patient for both treatments
